# PARADOXICAL ASSOCIATION BETWEEN AGING STEREOTYPES AND PROSOCIAL BEHAVIORS TOWARD OLDER ADULTS

**DOI:** 10.1093/geroni/igz038.2672

**Published:** 2019-11-08

**Authors:** Xin Zhang, Cai Xing, Ming Liu

**Affiliations:** 1 Peking University, Beijing, China; 2 Renmin University of China, Beijing, Beijing, China (People’s Republic); 3 Peking University, Beijing, Beijing, China (People’s Republic)

## Abstract

According to stereotype content model, older adults were perceived as low in competence (but high in warmth). Studies have demonstrated that such negative stereotypes could affect older adults significantly. However, it remained unclear how younger adults could be influenced, especially during intergenerational interactions, i.e., would positive or negative aging stereotypes promote more prosocial behaviors toward older adults. 104 younger adults were randomly assigned to three aging stereotype conditions (i.e., negative, neutral vs. positive), and they were then introduced to play two prosocial tasks (i.e., social value orientation and ultimatum game), in which they were imagined to play with either a younger or an older adults. It was found that younger adults exhibited more prosocial tendencies toward older partners than that to younger partners in both tasks. Moreover, activation of a negative aging stereotype could make younger adults behave more prosocially to the older partners in the social value orientation task.

